# Chronic inflammatory effects of in vivo irradiation of the murine heart on endothelial cells mimic mechanisms involved in atherosclerosis

**DOI:** 10.1007/s00066-023-02130-5

**Published:** 2023-09-02

**Authors:** Andrea Wittmann, Anna Bartels, Bayan Alkotub, Lisa Bauer, Morteza Hasanzadeh Kafshgari, Gabriele Multhoff

**Affiliations:** 1 Department of Radiation Oncology, School of Medicine, Klinikum rechts der Isar, Technische Universität München (TUM), Munich, Germany; 2 Center for Translational Cancer Research (TranslaTUM), School of Medicine Radiation Immuno-Oncology Group, Klinikum rechts der Isar, Technische Universität München (TUM), Ismaningerstr. 22, 81675 Munich, Germany; 3 Department of Nuclear Medicine, School of Medicine, Klinikum rechts der Isar, Technische Universität München (TUM), Munich, Germany; 4 Institute of Biological Medical Imaging, Helmholtz-Zentrum München (HMGU), Neuherberg, Munich, Germany; 5 Center for Translational Cancer Research (TranslaTUM), Heinz-Nixdorf-Chair for Biomedical Electronics, Klinikum rechts der Isar, Technische Universität München (TUM), Munich, Germany

**Keywords:** Thorax radiation, Lung microvasculature, Chronic/acute inflammation, Cardiac microvasculature, ICAM-1, Coronary disease

## Abstract

**Purpose:**

Radiotherapy is a major pillar in the treatment of solid tumors including breast cancer. However, epidemiological studies have revealed an increase in cardiac diseases approximately a decade after exposure of the thorax to ionizing irradiation, which might be related to vascular inflammation. Therefore, chronic inflammatory effects were examined in primary heart and lung endothelial cells (ECs) of mice after local heart irradiation.

**Methods:**

Long-lasting effects on primary ECs of the heart and lung were studied 20–50 weeks after local irradiation of the heart of mice (8 and 16 Gy) in vivo by multiparameter flow cytometry using antibodies directed against cell surface markers related to proliferation, stemness, lipid metabolism, and inflammation, and compared to those induced by occlusion of the left anterior descending coronary artery.

**Results:**

In vivo irradiation of the complete heart caused long-lasting persistent upregulation of inflammatory (HCAM, ICAM‑1, VCAM-1), proliferation (CD105), and lipid (CD36) markers on primary heart ECs and an upregulation of ICAM‑1 and VCAM‑1 on primary ECs of the partially irradiated lung lobe. An artificially induced heart infarction induces similar effects with respect to inflammatory markers, albeit in a shorter time period.

**Conclusion:**

The long-lasting upregulation of prominent inflammatory markers on primary heart and lung ECs suggests that local heart irradiation induces chronic inflammation in the microvasculature of the heart and partially irradiated lung that leads to cardiac injury which might be related to altered lipid metabolism in the heart.

**Supplementary Information:**

The online version of this article (10.1007/s00066-023-02130-5) contains supplementary material, which is available to authorized users.

## Introduction

Radiation remains indispensable for the treatment of solid tumors, with increasing insight into the properties of high-energy electromagnetic waves continuing to reveal new possibilities for its application in oncological diseases [1]. Although more than 50% of all solid tumors are treated with irradiation during cancer therapy [2–4], the healthy tissue surrounding the tumor is often also harmed. A comparison of the effects induced by exposure of the survivors of the Hiroshima and Nagasaki nuclear attacks with patients who received ionizing irradiation for peptic ulcers or breast cancer has revealed an increased relative risk of developing cardiac diseases with increasing radiation doses, irrespective of the radiation source [5]. The standard postoperative radiotherapy dose to the heart in women with breast cancer—the most common cancer in women worldwide [6]—ranges between 10 and 40 Gy [7]. It is now considered that acute and chronic cardiac pathologies such as coronary artery diseases, cardiomyopathy, and myocardial fibrosis that remain asymptomatic for nearly a decade [8–10] might be initiated by partial heart irradiation, which initiates irreversible heart failure [8, 9]. These findings are supported by randomized trials demonstrating a significantly increased risk for developing ischemic heart failure after postoperative radiotherapy in breast cancer patients [11]. Moreover, it has been reported that cardiac mortality is approximately 60% and 20% in patients with left- and right-sided breast cancer 10 years after irradiation, respectively [12], with the estimated mean dose to the heart after radiotherapy being 6.6 Gy and 2.9 Gy, respectively [13]. The linear increase in the risk of developing a major coronary event starting 5 years after the radiotherapy up to 20 years has been calculated to be 7.4% per Gy [13]. According to the cancer register, 22% of all deaths 10 years after thoracic irradiation are caused by heart diseases such as myocardial infarction and ischemic heart diseases [14], and of these 894 patients, 535 suffered from left-sided breast cancer.

The lung is also considered an organ at risk after irradiation of the thorax in breast cancer patients. Irrespective of the location of the breast cancer, several lobes of the lung are inevitably exposed to radiation. To avoid life-threatening radiation-induced lung diseases, the volume of the lung receiving up to 20 Gy (V20) should always remain below 10% and the mean dose to the lung should be lower than 6 Gy [15]. The close proximity of lung and heart cause reciprocal effects and multiorgan damage causes complications in both organs [16]. Radiation of one organ leads to a lower tolerated dose in the other [16], and co-irradiation of the heart and lung leads to drastically lower tolerated doses of both organs. Therefore, in this study, we investigated both primary heart and lung endothelial cells (ECs) derived from mice receiving complete local heart and partial lung irradiation.

Since recent studies have demonstrated that radiotherapy reduces the rate of relapses and breast cancer-mediated mortality rates after breast-conserving surgery by approximately 50%, radiotherapy is necessary and indispensable [17]. Previous data have demonstrated that vascular inflammation is an acute adverse radiation effect which contributes to normal tissue toxicity in the heart and lung [18]. The aim of this study was to identify and characterize long-lasting and late irradiation-induced effects in the microvasculature which might contribute to chronic heart diseases, including cardiac infarction. The CT-guided Small Animal Irradiation Platform (SARRP; Xstrahl, Camberley, UK) and a newly developed radiation plan enabled high-precision local irradiation of the heart, which spares large parts of the lung and thereby permits analysis of chronic, long-lasting irradiation effects (20 up to 50 weeks) in mice [18]. Herein, the chronic pathomechanisms of radiation on the microvasculature are compared to the acute effects induced by myocardial infarction.

## Materials and methods

### Computed tomography-guided irradiation of the heart and lung

Ten-week-old female C57Bl/6 mice (Charles River, Sulzfeld, Germany) were anesthetized by isoflurane/oxygen inhalation and randomly allocated to different treatment groups. Irradiation was performed using the high-precision image-guided Small Animal Radiation Research Platform (SARRP, Xstrahl, Camberley, UK). Briefly, cone beam computed tomography (CT; 60 kV, 0.8 mA) was performed to visualize the thorax in each mouse. For quantitative CT analysis, the region of interest (ROI) was manually inserted into the CT images of mice 20, 30, 40, and 50 weeks after irradiation with 0 (sham), 8, and 16 Gy. The ROI included the area of the heart exposed to the prescribed doses and excluded most parts of the lung to enable long-term survival of the mice up to at least 50 weeks. The heart was irradiated with 8 or 16 Gy (220 kV, 13 mA) using a lateral 6 × 8 mm^2^ X‑ray beam. Control mice were sham irradiated with 0 Gy but received a CT scan. The SARRP software and MuriPlan treatment planning system (Xstrahl) were used to precisely target the heart position and irradiation dose. The left lung lobe received an irradiation of less than 6 Gy and the right lung lobe remained unirradiated. The co-irradiation of the lung volume in total was 18% and the lung volume exposed to the maximum irradiation dose was 7%.

All animals were housed in individually ventilated cages (IVC) under specific pathogen-free conditions. All experiments were approved by the Government of Upper Bavaria and were performed in accordance with institutional guidelines of the Klinikum rechts der Isar, TUM, Germany.

### Immunohistochemical γH2AX staining

Irradiation delivery to the complete heart and to parts of the lung was determined with the DNA double-strand break marker γH2AX (Cell Signaling Technology, Danvers, MA, USA) on a Bond Rx staining machine (Leica Biosystems, Nussloch, Germany) using a Polymer Refine detection system without post-primary reagent. The heart and lung of one animal was removed 1 h after irradiation with 16 Gy, fixed in formalin overnight and embedded in paraffin. 2 µm sections were also stained with hematoxylin (Mayer’s hematoxylin) and eosin (0.5% aquaeous eosin γ‑solution).

### Left anterior descending coronary artery ligation

Ten-week-old female C57Bl/6 mice were anesthetized by intraperitoneal injection of midazolam (5 mg/kg body weight), medetomidine (0.5 mg/kg body weight), and fentanyl (0.05 mg/kg body weight). Mice were intubated and ventilated with oxygen-enriched air using a small animal respirator (MiniVent, Hans Sachs Elektronik, Germany). Buprenorphine (0.05 mg/kg body weight) was injected subcutaneously for analgesia. The thorax was opened by a 0.8 cm cut at the third intercostal space between the third and fourth ribs. The pericardium was removed and the left anterior descending artery (LAD) was permanently ligated for 15 weeks using an 8/0 monofilament polypropylene suture (PROLENE®, Ethicon, Norderstedt, Germany). Narcotics were antagonized using atipamezole (2.5 mg/kg body weight) and flumazenil (0.5 mg/kg body weight) via subcutaneous injection. Primary heart endothelial cells (ECs) were isolated from the infracted left heart ventricle and the noninfarcted right ventricle of the same mouse. The isolated primary ECs from these areas were screened for the indicated cell surface markers 15 weeks after the artificially induced heart infarction.

### Isolation of primary microvascular ECs from heart and lung

The isolation of primary ECs of mouse organs was performed as described previously [19]. Briefly, following craniocervical dislocation, the heart and left and right lung lobes of mice were collected under aseptic conditions. The left and right atria were surgically removed from the heart to reduce contamination with macrovascular ECs. After rinsing in ice-cold phosphate-buffered saline (PBS; Gibco/Thermo Fisher Scientific, Darmstadt, Germany) the tissues were minced with a sterile scalpel blade into cubes with a size of 1 mm^3^ and incubated in 10 ml of prewarmed (37 °C) digestion solution consisting of collagenase A (0.5 units/ml; Roche Penzberg, Germany) diluted in Hanks’ balanced salt solution (HBSS, Gibco)/10% v/v fetal bovine serum (FBS, PAA Laboratories GmbH, Freiburg i. Breisgau, Germany) for 45 min under gentle rotation (2 rpm). The suspension was forced 10 times through a sterile 18 G injection needle and filtered through a 70 µm cell strainer (BD Biosciences, Heidelberg, Germany). After two washing steps in 50 ml HBSS/10% v/v FBS solution (400 × g for 10 min), the final cell pellet was resuspended in 600 µl ice-cold isolation buffer (Invitrogen) with DSB‑X biotin-labeled (Molecular Probes) rat anti-mouse CD31 antibody (25 µl; 0.5 mg/ml; BD Biosciences, Heidelberg, Germany) for 10 min at 4 °C under gentle rotation (3 rpm). After another washing step in ice-cold isolation buffer, FlowComp™ beads coated with streptavidin (75 µl; Invitrogen/Thermo Fisher Scientific, Darmstadt, Germany) were added and incubated for another 15 min at 4 °C under gentle rotation. CD31-positive cells were isolated using a magnetic bead separation method (Invitrogen™ DynaMag™ magnet), after which CD31-positive cells immobilized to the DSB-X–streptavidin bead complex were released using biotin–streptavidin competition by incubating beads in 1 ml FlowComp™ release buffer (Invitrogen) for 2 min at 21 °C and pipetting 10 times. Isolated CD31-positive cells were used for flow cytometric analysis.

### Immunophenotypic characterization of primary ECs

Freshly isolated primary microvascular ECs were phenotypically characterized by flow cytometry on a BD FACSCalibur™ instrument (BD Biosciences, Heidelberg, Germany) using the following fluorescein (FITC)-, phycoerythrin (PE)-, or allophycocyanin (APC)- conjugated antibodies: integrin β3 (CD61, BD Biosciences, clone 2C9.G2), endoglin (CD105, eBioscience, clone MJ7/18), VE-cadherin (CD144, BD Bioscience, clone 11D4.1), mucosialin (CD34, eBioscience, clone RAM34), FAT (CD36, Invitrogen, 12-0362-82), PECAM‑1 (CD31, BD Biosciences, clone MEC 13.3), HCAM (CD44, Santa Cruz, sc-9960), ICAM‑1 (CD54, BD Biosciences, clone 3E2), ICAM‑2 (CD102, BD Biosciences, clone 3C4), VCAM‑1 (CD106, Santa Cruz, sc-18864), and common leukocyte marker (CD45, BD Biosciences, clone 30-F11) as a negative control. Appropriately labeled isotype-matched immunoglobulins were used as nonspecific binding controls. Briefly, 0.1 × 10^6^ viable cells were incubated with the indicated antibodies for 30 min at 4 °C in the dark. Following a washing step in PBS/FBS (10% v/v), cells were analyzed on a BD FACSCalibur™ instrument. Dead cells were excluded from the analysis by propidium iodide (PI) co-staining and a negative gating strategy.

### Statistical analysis

Data were analyzed using BD CellQuest™ Pro software (BD Biosciences) and the statistical significance of differences between experimental groups determined using Tukey’s test. Differences were considered significant at *p < 0.05 (5%), **p < 0.01 (1%), and ***p < 0.001 (0.1%). Data are presented as means of the number (*n*) of each experiment [19].

## Results

### Local CT-guided high-precision irradiation of the heart and lung of mice

**Fig. 1:**
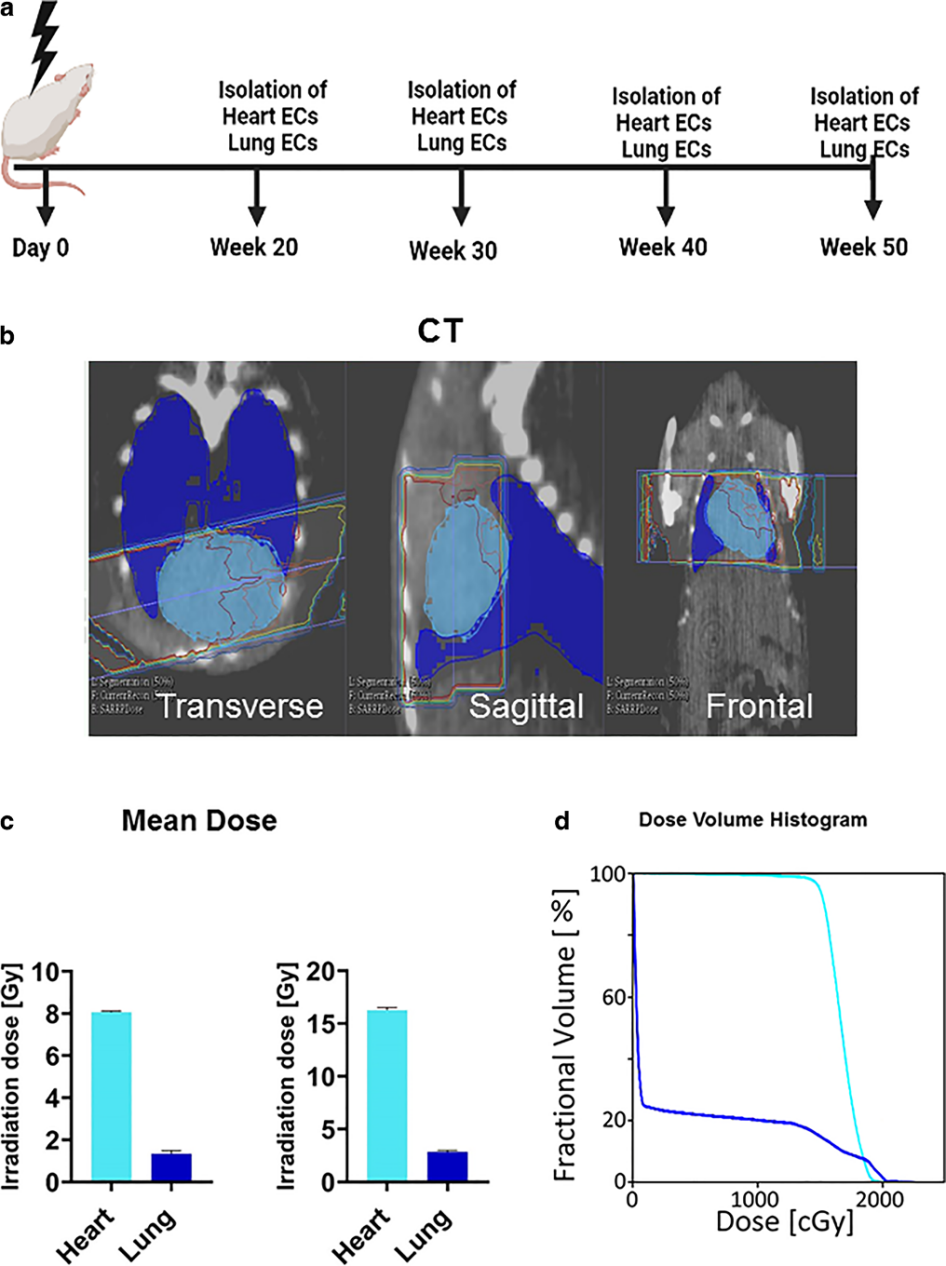
**a** Time schedule of the procedures and timepoints at which primary ECs were freshly isolated from mouse organs (heart, lung) after local in vivo irradiation of the complete heart and partial irradiation of the left lung lobe with 8 and 16 Gy. **b** Representative transverse, sagittal, and frontal views of CT images of a mouse thorax including the irradiation beamline. **c** Mean irradiation dose received by the heart and lung after a complete heart and partial lung irradiation with 8 and 16 Gy. **d** Representative dose–volume histogram of the heart and lung after a complete local heart and partial (less than 10% of the lung volume receiving 20 Gy) lung irradiation with 8 and 16 Gy

The cone beam computed tomography (CT) image-guided high-precision SARRP irradiated more than 95% of the heart volume and less than 10% of the lung with a dose of 8 and 16 Gy and enabled long-term survival of mice (C57Bl/6) up to at least 50 weeks. The right lobe of the lung remained unirradiated. All irradiated mice remained healthy up to 50 weeks and the treatment had no effect on their body weight (data not shown). The control group of mice was sham irradiated with 0 Gy. The time schedule and the applied procedures are schematically illustrated in Fig. 1a. For the irradiation, a beam diameter of 8 × 3 mm^2^ (composed of three overlapping single beams of 5 × 5, 3 × 3, and 3 × 3 mm^2^) was used [20]. The transverse, sagittal, and frontal view of the CT of a mouse thorax is illustrated in Fig. 1b, together with the mean dose (Fig. 1c) and dose–volume histogram (Fig. 1d) of the heart and lung after an irradiation with 8 and 16 Gy. The mean irradiation dose to the heart and the lung was 8.1 Gy vs. 1.3 Gy, and 16.2 Gy vs. 2.7 Gy, respectively (Fig. 1c). For high-precision local irradiation of the heart, the central axis of the beam was set to the isocenter of the heart using 220 kV and 13 mA X-ray irradiation with a copper filter (0.15 mm). The precision of the irradiation was confirmed by immunohistochemical staining of the irradiated and unirradiated tissues using the DNA double-strand repair protein marker γH2AX. For this experiment an additional mouse was sacrificed 1 h after exposure of the whole heart to 16 Gy because at later timepoints, γH2AX staining is no longer visible. The whole heart tissue but only part of the lung tissue showed positive foci after γH2AX staining (supplementary Fig. 1a, b).

### Long-term effects of in vivo irradiation of heart and lung

#### Effects on the percentage of cells expressing endothelial cell-related cell surface markers

**Fig. 2:**
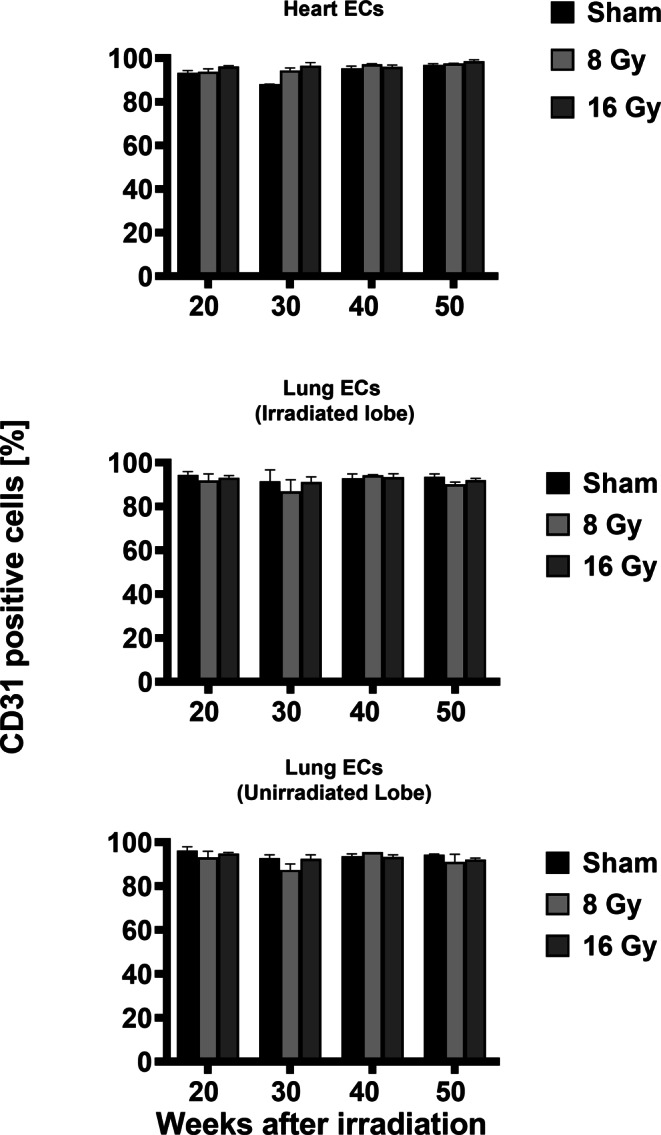
Percentage of cells stained positively with the markers CD31 (PECAM) on freshly isolated primary ECs after local in vivo irradiation of the complete heart and partial irradiation of the left lung lobe with 0, 8, and 16 Gy and the unirradiated right lung lobe of mice 20, 30, 40, and 50 weeks after treatment. Results represent mean values of three independent experiments with organs (heart, lung) of six mice at every timepoint and every radiation dose. Two organs were pooled for analysis. *ECs* endothelail cells

Primary ECs of the in vivo irradiated (sham 0, 8, 16 Gy) heart, left lung lobe, and unirradiated right lung lobe were isolated 20, 30, 40, and 50 weeks after irradiation [20]. Phenotypic characterization of the isolated primary ECs by flow cytometry involved analyzing the expression of cell surface markers that are typical for ECs such as PECAM‑1 (CD31), proliferation (integrin β3, CD61; endoglin, CD105; VE-cadherin, CD144), stemness (mucosialin, CD34), lipid metabolism (FAT, CD36), and inflammation (HCAM, CD44; ICAM‑1, CD54; ICAM‑2, CD102; VCAM‑1, CD106). A representative example of a gating strategy of the flow cytometric analysis of primary ECs derived from the heart of a mouse is shown in supplementary Fig. 2a–e. After a gating based on the size and granularity of the cells in an SSC(Side Scatter)-FSC(Forward Scatter) height plot (supplementary Fig. 2a), dead cells were excluded by PI staining (supplementary Fig. 2b). Isotype-matched control antibodies labeled with FITC, PE, and APC (supplementary Fig. 2c) were used to set gates for each fluorophore (supplementary Fig. 2d) to define the positively stained cell population (supplementary Fig. 2e). As shown in Fig. 2, isolated cells from 0 (sham), 8, and 16 Gy irradiated heart and lung tissues were all nearly 100% positively stained for the EC marker PECAM‑1 (CD31) at all measured timepoints (20, 30, 40, 50 weeks). A lower proportion of ECs isolated from heart and lung in the sham group was positively stained for the proliferation marker integrin β3 (CD61; mean 28 ± 4%), HCAM (CD44; mean 8 ± 6%), and VCAM‑1 (CD106; mean 29 ± 5%; supplementary Fig. 3). However, a significant increase in HCAM (CD44) positivity was observed on heart-derived ECs 20 (11 vs. 71%; *p* = 0.005), 30 (11 vs. 74%; *p* = 0.03), 40 (6 vs. 49%; *p* = 0.042), and 50 (5 vs. 38; *p* = 0.005) weeks after a local in vivo irradiation of the heart with 16 Gy (supplementary Fig. 3). Similarly, increases in positivity for VCAM‑1 (CD106), another inflammatory marker, of 39 to 51% (*p* = 0.015), 37 to 49% (*p* = 0.038), 38 to 52% (*p* = 0.036), and 33 to 53% (*p* = 0.033) 20, 30, 40, and 50 weeks after in vivo irradiation of the heart with 16 Gy were detected when compared to the sham group (supplementary Fig. 3). In contrast, ECs isolated from the unirradiated, sham irradiated, and partially irradiated left lung lobe showed no changes in the expression of HCAM (CD44; mean 54 ± 8%) and VCAM‑1 (CD106; mean 18 ± 0.3%) between 20 and 50 weeks after treatment (supplementary Fig. 3).

#### Effects on the density of expression of endothelial cell-related cell surface markers

**Fig. 3:**
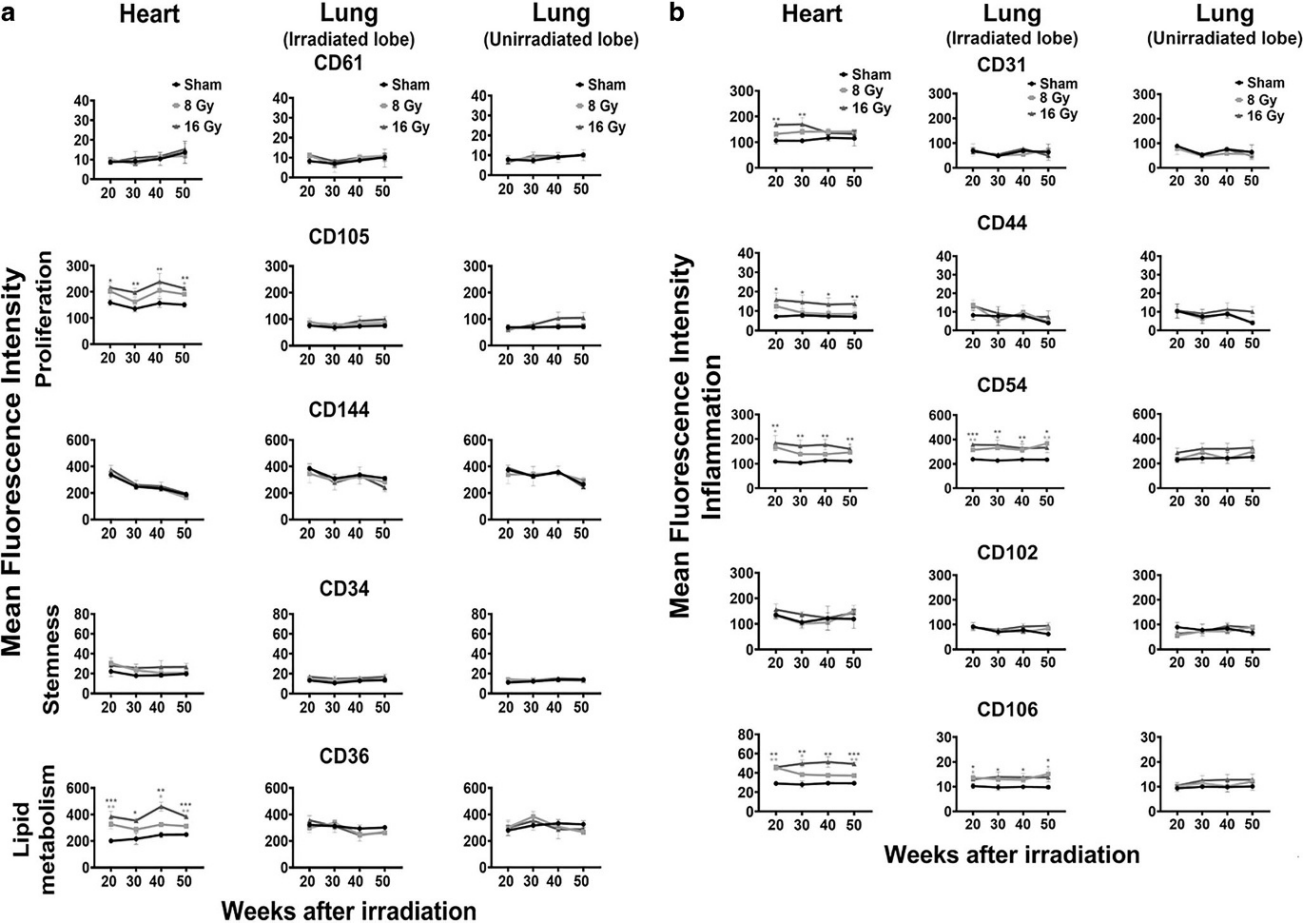
Mean fluorescence intensity (MFI) values of representative markers for proliferation (CD61, CD105, CD144), stemness (CD34), lipid metabolism (CD36; **a**) and inflammation (CD31, CD44, CD54, CD102, CD106; **b**) on freshly isolated primary ECs after local in vivo irradiation of the complete heart and partial irradiation of the left lung lobe with 0, 8, and 16 Gy and the unirradiated right lung lobe of mice 20, 30, 40, and 50 weeks after treatment. Results represent data of at least three independent experiments with organs (heart, lung) of at least six mice for every timepoint and irradiation dose. Two organs were pooled for analysis

The cell surface markers integrin β3 (CD61), endoglin (CD105), and VE-cadherin (CD144) are representative of the proliferative capacity of cells. Although the percentage of cells expressing the markers integrin β3 (CD61; supplementary Fig. 3) and VE-cadherin (CD105; data not shown) remained unchanged 20 to 50 weeks after in vivo irradiation of the heart and lung with 8 and 16 Gy, the density of the marker CD105 per cell, as determined by the mean fluorescence intensity (MFI) value, on heart ECs irradiated with 16 Gy was increased by approximately 50% (Fig. 3a). In contrast, the expression intensity of the proliferation markers on ECs isolated from partially irradiated lung tissues remained unaltered (Fig. 3a).

As a typical marker of the progenitor status, the expression density of mucosialin (CD34) was determined on primary ECs of the heart and lung 20, 30, 40, and 50 weeks after irradiation. Neither the percentage (data not shown) of ECs derived from heart and lung cells expressing CD34 nor its intensity of expression was significantly altered at all time points after in vivo irradiation of the organs with 8 and 16 Gy (Fig. 3a).

To determine the role of lipid oxidation/metabolism after irradiation, the expression density of the cell surface marker FAT (CD36) was analyzed on isolated primary ECs of the heart and lung*.* Irradiation of the heart with 8 and 16 Gy induced a significant increase in the MFI of CD36 expression on primary ECs of the heart 20 (8 Gy MFI: 201 vs. 326, *p* = 0.007; 16 Gy MFI: 201 vs. 385, *p* = 0.0009), 30 (16 Gy MFI: 216 vs. 354, *p* = 0.036), 40 (8 Gy MFI: 247 vs. 325, *p* = 0.043; 16 Gy MFI: 247 vs. 460, *p* = 0.001), and 50 (8 Gy MFI: 249 vs. 312, *p* = 0.002; 16 Gy MFI: 248 vs. 384, *p* = 0.0001) weeks after irradiation (Fig. 3a). In contrast, ECs isolated from the partially irradiated and unirradiated lung lobes or from the sham-irradiated organs exhibited no differences in the expression density of CD36 at any tested timepoint (Fig. 3a).

The expression densities of PECAM‑1 (CD31), HCAM (CD44), ICAM‑1 (CD54), ICAM‑2 (CD102), and VCAM‑1 (CD106) were measured to investigate irradiation-induced inflammation on primary ECs of the heart and lung. The MFI of CD31 expression on primary ECs isolated from the heart was transiently but significantly increased 20 and 30 weeks after irradiation of the heart with 16 Gy (20 weeks, *p* = 0.002; 30 weeks *p* = 0.003; Fig. 3b) and the CD44 expression density on heart-derived ECs was significantly increased at all measured timepoints after irradiation with 16 Gy (20 weeks, *p* = 0.01; 30 weeks, *p* = 0.02; 40 weeks, *p* = 0.02; 50 weeks, *p* = 0.001), as was the expression density of CD54 (20 weeks, *p* = 0.007; 30 weeks, *p* = 0.008; 40 weeks, *p* = 0.003; 50 weeks, *p* = 0.004; Fig. 3b). After irradiation with 8 Gy, CD54 expression on heart ECs was significantly increased after 20 (*p* = 0.023) and 50 weeks (*p* = 0.01). Although the percentage positivity of CD102 remained unaltered on heart ECs after in vivo irradiation of the heart, a long-lasting increase in the expression density of CD106 was observed on heart ECs after irradiation with 8 Gy (20 weeks, 8 Gy *p* = 0.0009; 30 weeks, 8 Gy *p* = 0.05; 50 weeks, 8 Gy *p* = 0.009) and 16 Gy (20 weeks 16 Gy *p* = 0.001; 30 weeks, 16 Gy *p* = 0.0009; 40 weeks, 16 Gy *p* = 0.001; 50 weeks 16 Gy *p* = 0.0006; Fig. 3b). CD106 (VCAM-1) and CD102 (ICAM-2) are both cellular adhesion molecules supporting leukocyte adhesion and therefore contribute to endothelial cell activation and inflammation by inducing the cytokine secretion of lymphocytes. CD102 as well as CD106 are abundantly expressed on vascular ECs and contribute to T cell adhesion/migration, NK cell cytotoxicity, and NK cell migration during ongoing inflammation, whereas the soluble form of CD106 promotes monocyte chemotaxis [21].

Primary ECs isolated from the partially irradiated left lung lobe also showed significant differences with respect to expression of the inflammation markers ICAM‑1 (CD54) and VCAM‑1 (CD106). After irradiation with 8 and 16 Gy, a chronic, long-lasting upregulated expression of CD54 was detected 20, 30, 40, and 50 weeks (20 weeks 8 Gy, *p* = 0.0011; 16 Gy, *p* = 0.00015; 30 weeks 8 Gy, *p* = 0.015; 16 Gy *p* = 0.004; 40 weeks 8 Gy, *p* = 0.02; 16 Gy, *p* = 0.008; 50 weeks 8 Gy, *p* = 0.005; 16 Gy *p* = 0.02). The expression density of CD106 on ECs increased significantly after partial irradiation of the left lung with 8 Gy after 20 and 50 weeks (20 weeks 8 Gy, *p* = 0.018; 50 weeks 8 Gy, *p* = 0.025) and was permanently increased between 20 and 50 weeks after irradiation with 16 Gy (20 weeks 16 Gy, *p* = 0.043; 30 weeks 16 Gy, *p* = 0.025; 40 weeks 16 Gy, *p* = 0.03; 50 weeks 16 Gy, *p* = 0.; Fig. 3b). The phenotype of ECs isolated from the unirradiated lung lobe was neither affected by irradiation of the other lung lobe nor by complete local heart irradiation.

### LAD-induced heart infarction

#### Effects on the density of cell surface marker expression by primary heart ECs

**Fig. 4:**
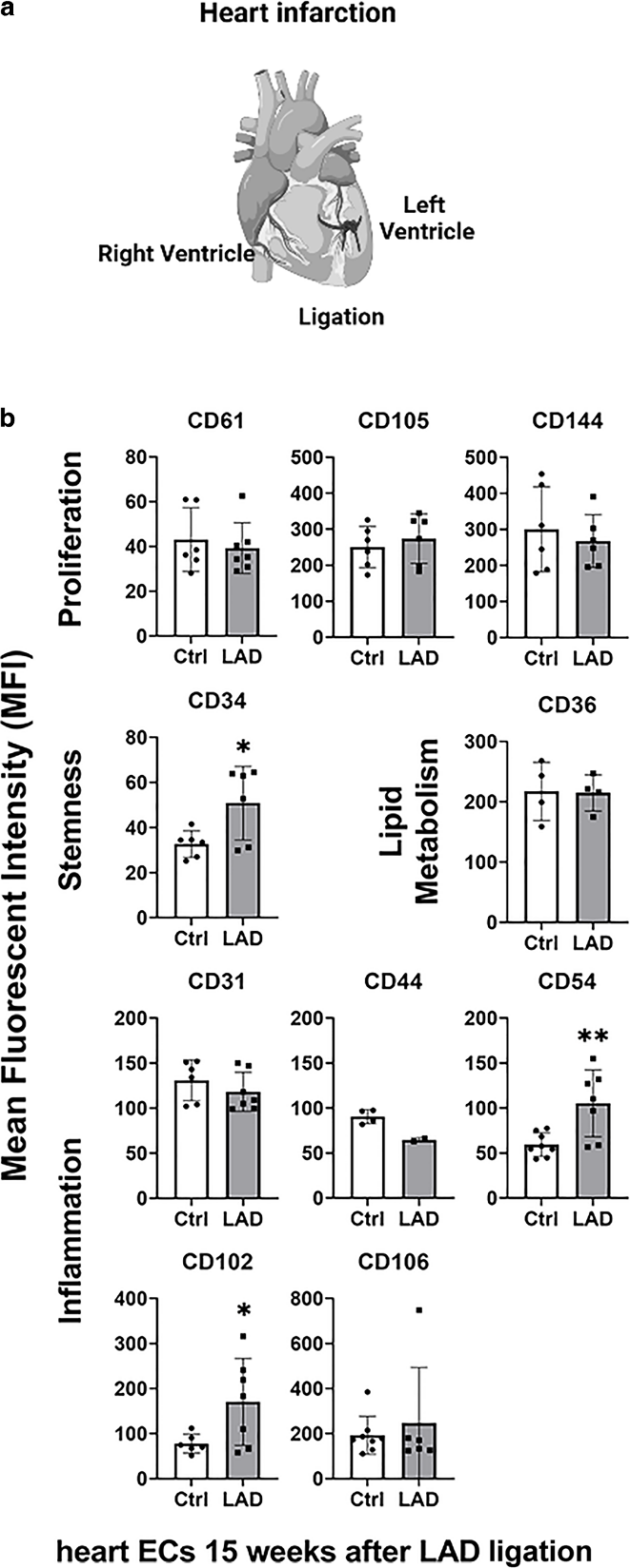
**a** Schematic representation of a heart after ligation of the left anterior descending coronary artery (LAD). *Arrows* indicate the infarcted (left ventricle) and noninfarcted (right ventricle) heart areas from which primary ECs were isolated. **b** Mean fluorescence intensity (MFI) of proliferation (CD61, CD105, CD144), stemness (CD34), lipid metabolism (CD36), and inflammatory (CD31, CD44, CD54, CD102, CD106) markers on freshly isolated primary ECs derived from infarcted and noninfarcted control heart areas 15 weeks after LAD ligation. Data represent results from hearts of six animals 15 weeks after LAD ligation with 3 to 8 measurements. Two hearts were pooled for analysis

Primary heart ECs were isolated and screened for expression of the different markers before 15 weeks after an artificially induced heart infarction (occlusion of the LAD). ECs were isolated form the infarcted left heart ventricle and from the noninfarcted right heart ventricle (control) of the same mouse (Fig. 4a). As shown in Fig. 4b, none of the markers characteristic for proliferation (CD61, CD144, CD105) were altered on ECs isolated from the infarcted left ventricle, although chronic inflammation after irradiation induced upregulation of the marker CD105. This discrepancy might be due to differences in the severity of the damage induced by heart infarction in the left ventricle or ionizing irradiation to the complete heart. However, similar to the chronic effects induced by irradiation, the MFI value of the most prominent inflammatory marker CD54 was significantly (CD54, *p* = 0.006) increased on ECs derived from the infarcted tissue compared to the noninfarcted control tissue (Fig. 4b). Although CD106 mediating adhesion of immune cells to the vascular endothelium during inflammation [21] is highly upregulated by irradiation, this marker was only moderately increased by LAD occlusion (CD106, *p* = 0.573). This might be explained by differences in the duration of inflammation after LAD occlusion and irradiation. A significant upregulation of CD102 (CD102, *p* = 0.042) by LAD occlusion, which indicates increased vascular T cell adhesion, NK cell cytotoxicity, and migration [21], might also contribute to inflammation.

In addition to the inflammatory markers, the stemness marker mucosialin (CD34) was found to be significantly elevated on ECs derived from the infarcted tissue (CD34, *p* = 0.028), which might represent a compensatory effect of the acutely damaged ECs (Fig. 4b).

## Discussion

Although irradiation is an indispensable treatment option for many solid tumors, chronic toxicity to normal tissues can limit the therapeutic success. High-dose irradiation of breast, lung, or esophageal cancer can cause chronic heart failure approximately a decade after radiation therapy [22]. In order to better understand the long-term consequences of irradiation in this context, we have studied the chronic effects (20 to 50 weeks) of local heart irradiation and partial irradiation of the left lung lobe on the biology of primary ECs isolated from these organs in mice. The parameters that were examined—proliferation, stemness, lipid metabolism, adhesion, and inflammation—are known to increase the risk for developing chronic cardiac diseases such as myocardial infarction after irradiation of the left thorax in breast cancer patients [5]. Although previous studies have described acute inflammatory responses of normal tissues of mice after irradiation [23], chronic effects were not studied due to the limited overall survival of mice after complete chest irradiation [20]. Our laboratory has developed a radiation plan for a high-precision local heart irradiation that spares large parts of the lung tissue and allows much longer survival of the mice [18]. In a previous study, inflammatory and fibrotic changes in the irradiated heart and lung tissue 50 weeks after local heart irradiation of mice was demonstrated by the existence of intra-alveolar foam cells, heart hyaline deposition, lung interstitial fibrosis, lung perivascular infiltrations, and an increased lung density as determined by cone beam CT [20]. Herein, we have demonstrated for the first time a chronic and long-lasting upregulated expression of major inflammatory markers such as CD44 (HCAM), CD54 (ICAM-1), and CD106 (VCAM-1) on primary heart ECs up to 50 weeks after local heart irradiation with clinically relevant doses of 8 and 16 Gy. These data indicate an ongoing chronic inflammatory process in the heart microvasculature as a late side effect after heart irradiation which might attract leukocytes to the endothelium and thereby promote further damage of the vessels [24]. Chronic microvascular dysfunction induce complications in vascular tone and blood hemostasis, and promote chronic inflammatory processes [25] which, in turn, induce radiation-induced cardiovascular injuries [9]. Ischemic heart disease is the most common cause of death after radiotherapy [26]. A long-term and permanent upregulation of inflammatory markers generates a chronic inflammatory micromilieu which accelerates atherosclerotic lesions in coronary vessels, pericardial fibrosis, myocardial fibrosis, and vessel calcination [8].

Local irradiation of the heart with 8 Gy also caused permanent upregulation of CD36 (FAT), an integral membrane glycoprotein regulating lipid metabolism. CD36 is responsible for the uptake of cholesterol by macrophages and supports their transformation into lipid-loaded foam cells [27] which are involved in the formation of atherosclerosis-like lesions in the microvasculature [28, 29]. The removal of cholesterol from foam cells is regulated by the transcription factor peroxisome proliferator-activated receptor alpha and gamma (PPAR‑α, PPAR-γ), which inhibits lipid-metabolizing enzymes [30, 31]. As the activity of PPAR‑α after radiotherapy appears to be drastically decreased [32] 8 and 40 weeks after irradiation [33], cholesterol removal is prevented and the synthesis as well as uptake of fatty acids might therefore be increased. An increased formation of foam cells can induce chronic inflammation which promotes the onset of atherosclerosis [34, 35], but the disease remains asymptomatic for nearly a decade before the cardiovascular disease-related mortality in patients increases significantly [36, 37] after radiation of left-sided breast tumors [38]. This implies that several diseases like obstructive coronary artery disease, myocardial fibrosis, pericardial disease, arrhythmias, and valvular abnormalities are most likely related to a previous thoracic radiotherapy [9]. The apex and the anterior wall of the heart are the anatomic sites that obtain the highest radiation doses. Therefore, the risk of developing radiation-induced diseases such as atherosclerosis leading to myocardial infarction years later is very high, especially in this part of the heart [39, 40]. Depending on dose and duration of the radiation therapy, the risk of developing coronary artery diseases increases significantly [36].

In the present study, some of the main late occurring side effects of irradiation of the heart, such as EC dysfunction leading to chronic inflammation and the development of atherosclerosis, could be documented on primary ECs of the heart and lung in a mouse model.

Ligation of the LAD is used as a model for myocardial infarction in mice [41]. Interestingly, an artificially induced heart infarction in mice also causes a significant increase in the expression of prominent inflammatory markers CD54 (ICAM-1), CD102 (ICAM-2), and CD106 (VCAM-1) on primary heart ECs of the infarcted area compared to those of the noninfarcted area of the same heart. This may suggest that chronically induced inflammatory effects caused by a local heart irradiation on primary microvascular ECs also play a role in response to a heart infarction. Hence, acute inflammatory processes after myocardial infarction are similar to long-term inflammatory effects after radiotherapy. Therefore, we speculate that strategies preventing acute and chronic inflammation of the microvasculature in the heart might help to avoid cardiovascular complications. One strategy to avoid inflammation involving lipid metabolism might be the re-activation of PPAR‑α by fenofibrate, which decreases foam cell formation and might thereby help to decrease cardiac inflammation [30].

It is known that the interaction between heart and lung influences the total tolerated radiation dose. The radiation of one organ leads to a lower tolerated dose in the other organ [16]. This effect is responsible for tachypnoea and right ventricular hypertrophy after radiation with doses above 20 Gy for the heart and 5 Gy for the lungs [42–44]. As the highest radiation dose in the described experiments was 16 Gy for the heart and 3 Gy for the lung, the reciprocal effect between heart and lung tissue after radiation appears to be less relevant under these conditions and, therefore, long-term survival of the mice was maintained, which enabled us to study chronic irradiation effects. Our data might suggest that chronic negative abscopal effects induced by complete heart and partial lung irradiation with 16 Gy are unlikely, since in our mouse model, the nonirradiated lung ECs remained unaffected up to 50 weeks after irradiation.

### Supplementary Information

Supplementary Fig. 1a. Immunohistochemical staining of γH2AX of the irradiated heart tissue 1 h after irradiation with 16 Gy. γH2AX foci (*brown dots*) are visible throughout the whole tissue. Scale bar 50 µm.

Supplementary Fig. 1b. Immunohistochemical staining of γH2AX of the partially irradiated lung tissue 1 h after irradiation with 16 Gy. γH2AX foci (*brown dots*) are visible in the partially irradiated part of the lung tissue (right), but not in the unirradiated tissue (left). Scale bar 50 µm.

Supplementary Fig. 2. Representative example of a gating strategy for the flow cytometric analysis of freshly isolated primary ECs of the heart. a) SSC-FSC height plot gating separate cells from debris. b) Exclusion of dead cells by propidium iodide (PI) staining. c) Staining of cells with isotype-matched control antibodies labeled with FITC, PE, and APC. d) Exclusion of lymphocytes by negative gating of CD45-positively stained cells. e) Examples of a CD34-FITC and CD144-PE staining of primary ECs.

Supplementary Fig. 3. Percentage of cells stained positively with the markers CD61 (integrin β3), CD44 (HCAM), and CD106 (VCAM) on freshly isolated primary ECs derived from the heart and lung (partially irradiated left lung lobe, unirradiated right lung lobe) 20, 30, 40, and 50 weeks after a local heart irradiation. Results represent mean values of the organs of three mice.
