# Supplementary figures and images for: Chronic inflammatory effects of in vivo irradiation of the murine heart on endothelial cells mimic mechanisms involved in atherosclerosis
Source: Strahlenther Onkol. 2023 Sep 2;199(12):1214–24. doi: 10.1007/s00066-023-02130-5 (PMC10673733; doi:10.1007/s00066-023-02130-5)

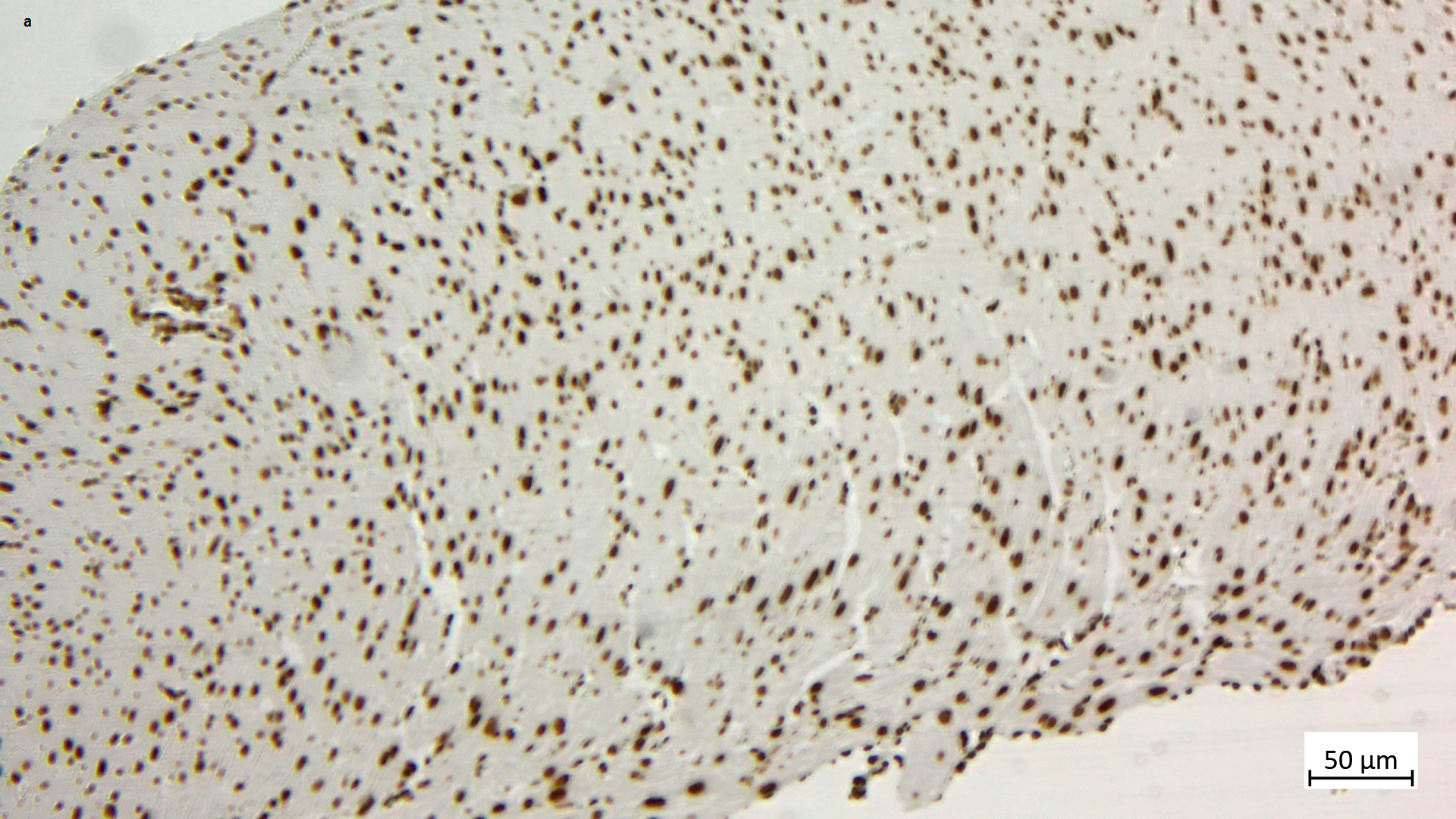

Supplement: Supplementary file 1 — Supplementary Fig. 1a. Immunohistochemical staining of γH2AX of the irradiated heart tissue 1 h after irradiation with 16 Gy. γH2AX foci (brown dots) are visible throughout the whole tissue. Scale bar 50 µm. [file 66_2023_2130_MOESM1_ESM.tif]

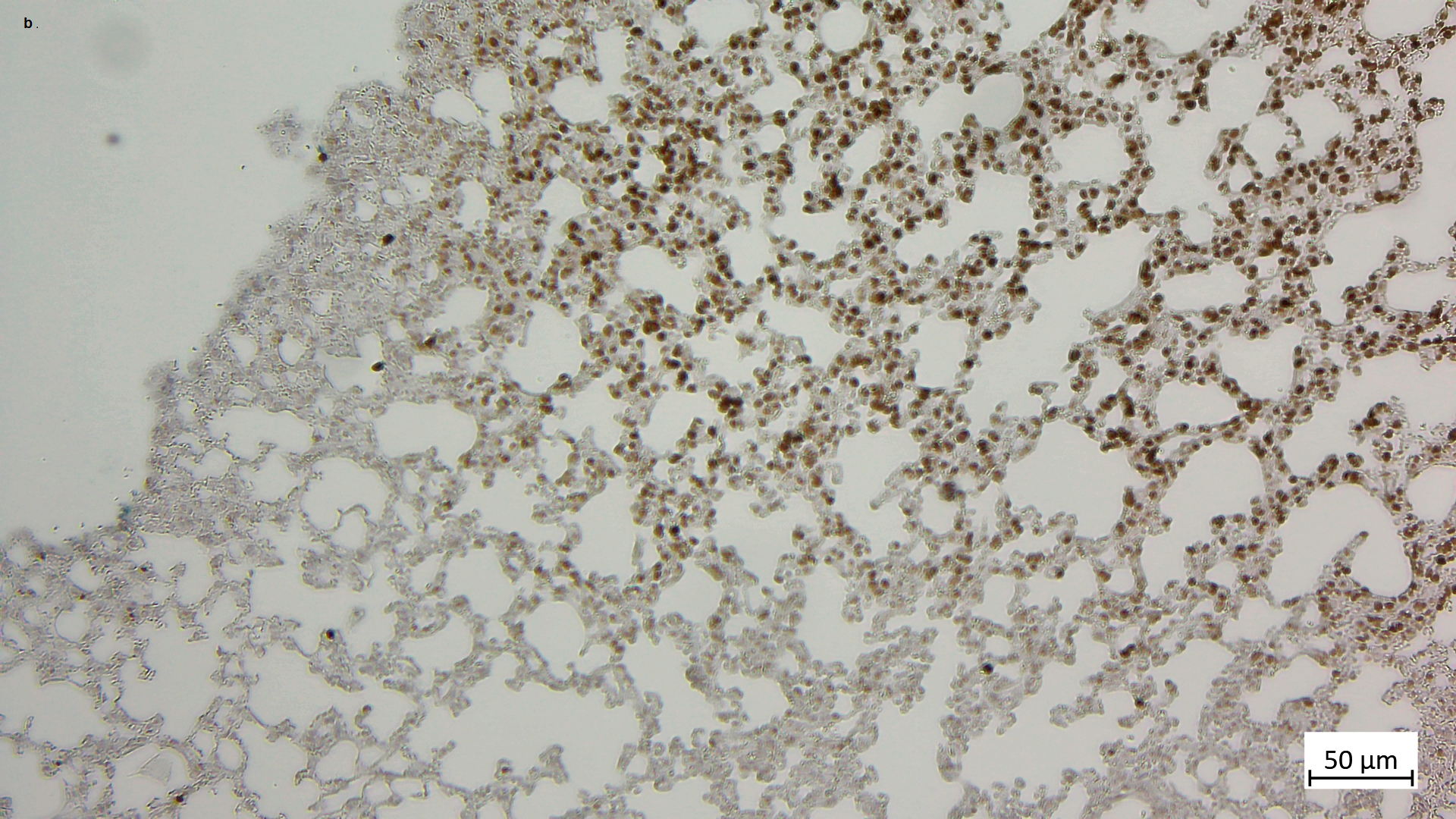

Supplement: Supplementary file 2 — Supplementary Fig. 1b. Immunohistochemical staining of γH2AX of the partially irradiated lung tissue 1 h after irradiation with 16 Gy. γH2AX foci (brown dots) are visible in the partially irradiated part of the lung tissue (right), but not in the unirradiated tissue (left). Scale bar 50 µm. [file 66_2023_2130_MOESM2_ESM.tif]

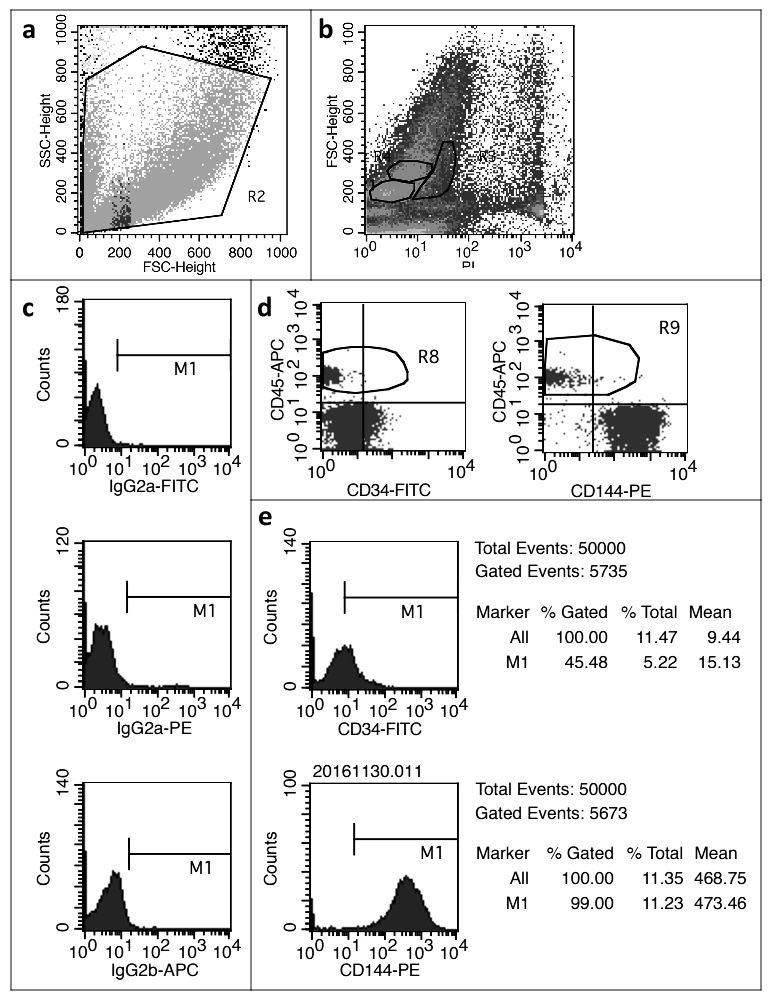

Supplement: Supplementary file 3 — Supplementary Fig. 2. Representative example of a gating strategy for the flow cytometric analysis of freshly isolated primary ECs of the heart. a) SSC-FSC height plot gating separate cells from debris. b) Exclusion of dead cells by propidium iodide (PI) staining. c) Staining of cells with isotype-matched control antibodies labeled with FITC, PE, and APC. d) Exclusion of lymphocytes by negative gating of CD45-positively stained cells. e) Examples of a CD34-FITC and CD144-PE staining of primary ECs. [file 66_2023_2130_MOESM3_ESM.tif]

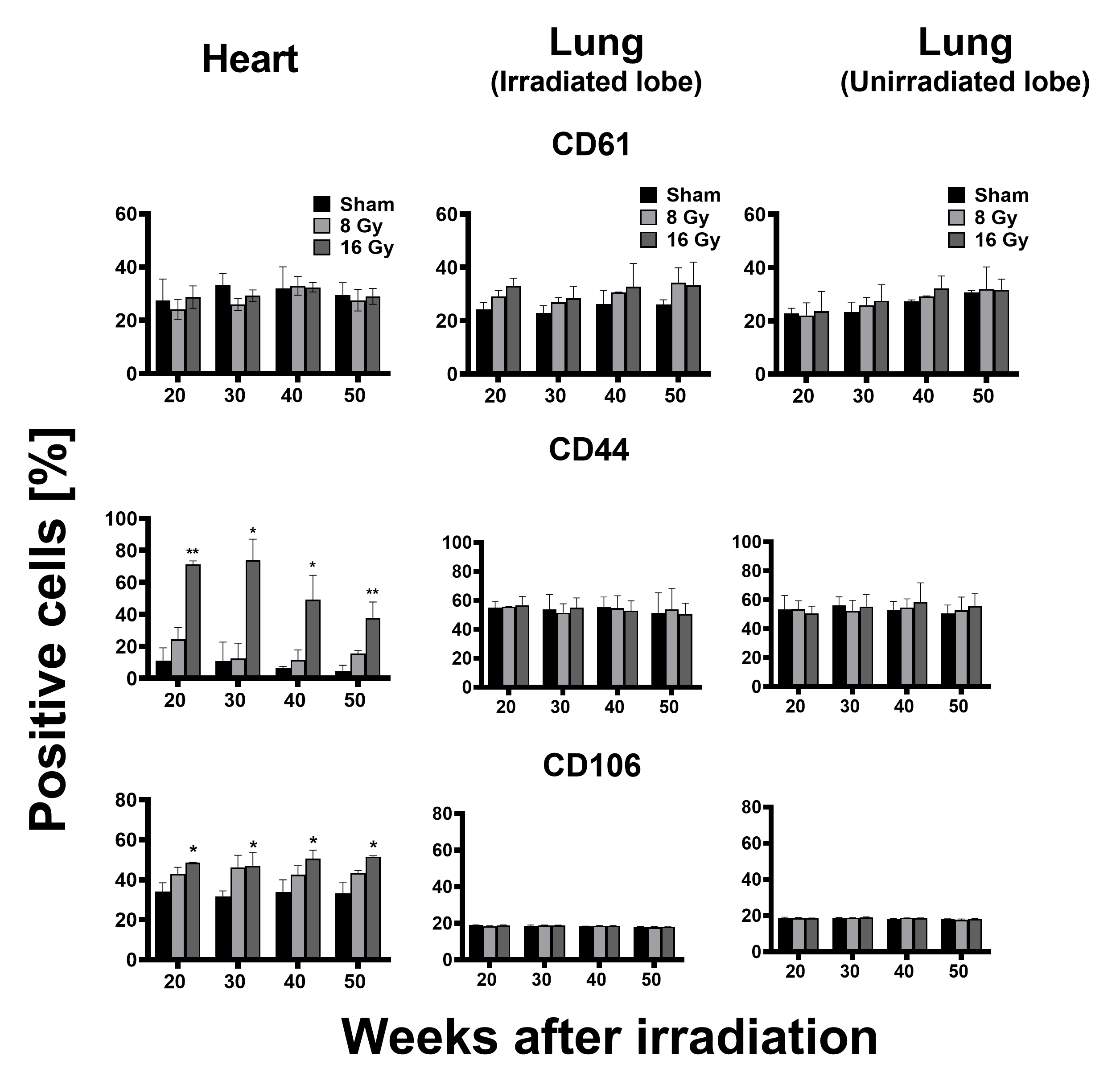

Supplement: Supplementary file 4 — Supplementary Fig. 3. Percentage of cells stained positively with the markers CD61 (integrin β3), CD44 (HCAM), and CD106 (VCAM) on freshly isolated primary ECs derived from the heart and lung (partially irradiated left lung lobe, unirradiated right lung lobe) 20, 30, 40, and 50 weeks after a local heart irradiation. Results represent mean values of the organs of three mice. [file 66_2023_2130_MOESM4_ESM.tif]
